# Supplementary material for: Predicting knee adduction moment response to gait retraining with minimal clinical data
Source: PLoS Comput Biol. 2022 May 16;18(5):e1009500. doi: 10.1371/journal.pcbi.1009500 (PMC9135336; doi:10.1371/journal.pcbi.1009500)
Supplement: Attachment — Submitted filename: ReviewerResponses_PLOSCompBio2022_draft_v6.docx [file pcbi.1009500.s001.docx]

Dear Reviewers, Dr. Dingwell, and Dr. Beard,

We thank you for your careful consideration of our manuscript. We have responded to all of your comments below and have made changes to the manuscript accordingly. New manuscript text is quoted after each response, and all modifications are highlighted in the marked-up version of our revised manuscript. The last section of this document summarizes the additional changes that we made during the revision process. We agree with your insightful suggestions and believe they have notably strengthened this revision.

We appreciate your time and effort and look forward to hearing from you.

**Responses to Reviewer #1:**

1. Abstract: Provides a good overview, but one key piece of information is missing – detail on the model itself. Just an extra sentence or phrase giving the reader some idea of what kind of model it is, what it involves (input, etc.).

We have added information on the model to the fifth sentence of the abstract (lines 7-8), which we agree will help the reader understand a key detail of our work.

“We present a regression model that uses minimal clinical data — a set of six features easily obtained in the clinic — to predict the extent of first peak KAM reduction after toe-in gait retraining.”

2. Lines 37-38, ‘medial compartment’: is the medial compartment of the knee generally the one to be more problematic in OA? It might be beneficial, for the broad readership of PLOS Comp Biol, to add some extra detail/justification on this point.

Yes, this is an important justification to add. Osteoarthritis occurs more commonly in the medial compartment of the knee. We have added these details to the Introduction (lines 34-36) along with the relevant citations.

“Osteoarthritis presents more often in the medial compartment than the lateral compartment of the knee partially because the common varus (bow‑leg) alignment increases medial knee contact force [3,4].”

3. Line 47, “Walking with the toes pointed inward”: I recommend moving this phrase up two sentences, immediately after when FPA is first introduced. This should help with clarity for the non-specialist.

We have made the suggested change (lines 43-45).

“Gait modification strategies to reduce KAM have included decreasing walking speed [10,11], increasing trunk sway [10,12-15], and changing the foot progression angle (FPA) by walking with the toes pointed inward or outward [16-21].”

4. Lines 87-88, “The last ten strides…”: Do you mean strides or steps here? Later on you talk about steps. A single stride comprises two steps. Also, this suggests that both right and left legs are considered in the study, which would be worth explicitly clarifying.

Thank you for pointing out this error. We have changed “strides” to “steps” and added a phrase clarifying that only data from the affected leg were used to learn toe-in gait patterns (line 97-98).

“The last ten steps of the osteoarthritic leg in each condition were used for analysis.”

5. Line 130, “landing cleanly on the force plates”: Isn’t this inconsistent with the use of a treadmill? Some extra clarification is required here.

We use force plate data collected with instrumented split-belt treadmills which have two force plates. Sometimes subjects will step on the edge of a force plate. We have added clarification that this is a “split-belt” treadmill (line 139) and also clarified that the two force plates are in the treadmill (line 141). We hope these additions will help clarify any confusion.

“Subjects walked at a self-selected speed on an instrumented split-belt treadmill, and data were collected for approximately 2 minutes. We filtered ground reaction force data at 15 Hz using a fourth‑order zero‑lag Butterworth low‑pass filter and removed steps that did not land cleanly on the treadmill’s two force plates.”

6. Line 144: I presume that the ‘static alignment’ is measured when the subject is standing still, correct? An extra clarifying phrase would help here.

This is correct. We have added a clarification to lines 151-153.

“The static knee alignment was taken when the subject was standing still and was defined as the angle between the vectors connecting 1) the lateral malleolus and lateral epicondyle and 2) the lateral epicondyle and hip joint center, in the frontal plane, with valgus angle defined as positive.”

7. Fig. 1: Excellent figure. Are panels (B) and (C) stylistic only, or are they based on actual data? If the latter, this should be explained/cited in the caption as appropriate. Also, the description for panel (A) should use “external moment” instead of just “moment”.

Thank you. Yes, panels (B) and (C) in Figure 1 are indeed based on actual data; they are group averages from the Stanford dataset, a citation that we have now added to the caption. We have added “external” to the knee adduction moment label and have also made other stylistic changes to improve this figure.

8. Fig. 3: I am curious as to why the trajectory for the 10 degree bin is so markedly different from the trajectories of the other bins. Is this because of a small sample size or outlier at this extreme foot position? And how much does this affect your model? Some discussion of this, in the appropriate part of the text, would be welcomed.

Yes, at 10° toe-in, the mediolateral knee joint center position offset differs from the trajectory at other angles, particularly in the last half of stance. This may be due to a small sample size at this foot angle (6 steps) compared to others (21 steps at 5-degrees, for instance). We have added a sentence to the Methods section indicating the range of the number of steps in each bin (lines 113-114).

“The number of steps in each bin ranged from 6 in the 10° bin to 21 in the 5° bin.”

We do not expect that this trajectory significantly impacted the predictive model, as the first peak of KAM occurs in the first half of stance, where the 10-degree bin trajectory is more similar to that of the other bins. Additionally, this is only one of four parameters (including anterior-posterior knee joint center, mediolateral and anterior-posterior center of pressure) used to estimate KAM. Given that the predicted knee joint center positions were more accurate in the mediolateral direction than in the anterior-posterior directions, for both the Stanford University and the Carnegie Mellon University datasets, we have confidence that the model was not impacted by this bin’s smaller sample size. We have added further detail to lines 114-117 to contextualize our use of fitting splines to the averaged trajectories in order to smooth the learned gait patterns:

“Trajectories were averaged within each bin. We represented each of the averaged trajectories using basis splines of order 12 to smooth the prediction. This order was selected from a range of 3 to 20 via cross-validation; increasing beyond 12 yielded no further reduction in root mean squared error (RMSE) between the trajectory and the spline.”

9. Fig. 5: In the left panel, I recommend indicating that the dashed line is a line of parity, not some fitted regression line. The same goes for Fig. 7 as well.

Thank you for this suggestion. Although this line was initially a line of parity, we have changed it to the line of best fit in order to provide more detail on the regression model’s accuracy. We have added information on the R^2^ values between the actual first peak KAM reduction and the predicted KAM reduction to the Results section (lines 204 and 239). We have also added that the dashed line is now the line of best fit in the legends of Figures 5 and 7:

Figure 5: “The line of best fit (dashed line) has an R^2^ of 0.87.”

Figure 7: “The line of best fit (dashed line) has an R^2^ of 0.55.”

**Responses to Reviewer #2:**

1. The introduction effectively reports the clinical overview, describing the correlation between higher peaks of KAM and osteoarthritis progression, the possible non-invasive therapies by changing the foot progression angle, and the need of automating the procedure of target foot progression angle's evaluation. However, a better overview of previous data-driven studies addressing similar problems in gait biomechanics would be necessary. It would be very interesting to highlight previous contributions with their limitations and clearly state which of these limitations this study aims to overcome. A study is mentioned in line 291 in the Discussion (reference 36), but a more extensive state-of-the-art would better define this manuscript's contribution and novelty. For instance, a recent study used a feedforward neural network to predict ground reaction forces and joint moments (https://doi.org/10.1016/j.medengphy.2020.10.001). Another one used a neural network to predict KAM from anatomical landmarks obtained in 2D video analysis (https://doi.org/10.1016/j.joca.2020.12.017). There are likely other publications about the use of data-driven approaches in biomechanics that would be interesting to compare.

Thank you for this suggestion. Our work seeks to predict KAM reduction in response to toe-in gait, whereas prior work has focused on predicting KAM with emerging wearable sensors (e.g., inertial measurement units and video cameras). While KAM reduction can be computed using prior KAM estimation models, the accuracy of these prior models is lower than what is required to determine whether a patient will benefit from gait retraining therapy or not. Our KAM reduction model has a MAE of 0.134 %BW*HT on actual test set data, while prior KAM estimation methods report MAEs of 0.37–0.49 %BW∗HT (Boswell et al., 2021).

Most importantly, our model can predict KAM reduction to this accuracy without using any equipment. The only required measurements are height, weight, walking speed, limb alignment, and FPA. In contrast, other methods still require some gait analysis, albeit with more minimal equipment than a full gait laboratory. For example, the model of Boswell et al. (2021) predicts KAM using gait analysis from video data, but it has not been validated with real video data, as it was trained and tested entirely on synthetic data generated from optical motion capture. The model of Mundt et al. (2021) predicts KAM from marker data, which is not feasible to collect in the clinic.

We agree that the study by Boswell et al. (2021) (formerly reference 36, now 28) is of particular significance. We had described the relevance of this study to our work in the Discussion section but now include a reference to it in the Introduction, along with a brief overview of other studies (lines 65-68), including work by Mundt et al. that contrasts several machine learning approaches for predicting KAM from wearable sensors [27]. We highlight that our model (1) targets a different task (prediction of KAM reduction in response to gait retraining interventions, line 68); (2) achieves better accuracy (line 312); (3) does so using no gait analysis equipment (lines 318-320); and (4) is cautiously validated on independent data, in addition to the traditional approach of validating on left-out test data from the same dataset (lines 258-260). In addition to the clinical utility, we believe that this level of rigor also sets our model apart.

Introduction

“At present, researchers must iteratively evaluate KAM reduction at a range of foot progression angles, relying on force plates and motion capture to compute and compare joint moments. Although proposed methods for estimating KAM using wearable sensors [26, 27] or synthetic video data [28] could help reduce reliance on gait lab equipment, these approaches still require gait data collection. No methods currently exist to automate the procedure of predicting KAM reduction in response to gait retraining interventions.”

Discussion

“In a previous study by Boswell et al. [28], a neural network trained on time discretized 3D anatomical features from 86 subjects was used to classify whether an individual would increase or reduce their first peak KAM with toe in or toe out gait modifications, attaining accuracies of up to 85%. Their models predicted the peak KAM during baseline walking with MAE ranging from 0.37–0.49 %BW∗HT. The most salient features of Boswell et al.’s model included those related to the position of the pelvis, knee angle in the frontal plane, and sway of the trunk; in the future, a sensor fusion approach that combines wearable sensing and vision based motion capture could make use of these additional pelvis and trunk features to improve our predictive model. However, while our model was capable of correctly predicting a KAM reduction for all subjects in the external dataset, we sought to estimate the extent of KAM reduction, rather than classify subjects based on whether KAM would decrease or not. Furthermore, estimating KAM reduction from minimal clinical data, as we have done here, instead of computing KAM from cameras or wearable sensors [26, 27], eliminates the need for gait analysis. A fast estimate of the KAM reduction with gait retraining may empower clinicians to weigh the expected benefit of retraining against that of other treatment alternatives.”

We hope that these changes help to better situate our contribution within the current research landscape.

2. The methodology is rigorous and very well explained. A small clarification could be done in the motivation to choose the six features. Why are these features chosen and not others? Is it just because they are easy to measure? How is the choice of these features more advantageous compared to other studies? (see previous links and https://doi.org/10.1016/j.knee.2020.12.006)

Yes, we use these six features in the model because they are easy to measure and relevant to KAM. These features are either commonly collected in the clinic (height, weight, static alignment), directly contribute to the calculation of the KAM (ground reaction force), or have been previously shown to correlate with KAM (toe-in angle). We had motivated this in the last paragraph of the introduction (lines 70-71) but believe that our added clarification to the abstract (lines 7-8) will help reinforce this point. We agree that the features described in previous studies, such as those extracted from synthetic video data [28], could be used to improve a predictive model; this is an exciting prospect for future work that we discuss in lines 314-316. Also, the work of Lindsey et al. meaningfully contributes to the existing literature on the relationships between foot progression angle changes and medial knee thrust when changing gait to reduce KAM. We are grateful for the suggestion to reference their work in our manuscript ([21]) and have added this citation to our Introduction in line 45. However, this study focuses on identifying shared mechanisms between different gait modification strategies (toe in and trunk sway), rather than identifying easy-to-measure features that are predictive of response to gait retraining, and therefore it does not directly inform our selection of predictive features.

3. The Results are detailed and promising, and the Discussion illustrates well the main achievements and the limitations of the study.
   3.1 The Results section ranks the six features from the most significant to the lowest (line 201-206). Successively, it has been verified that the toe-in angle alone could predict the KAM reduction with a small error range. Could the authors discuss their recommendation concerning how many and which features they would use, and briefly give a reason?

Yes – although we mention in the discussion that using the full set of six features results in the lowest MAE (lines 291-292), we have now added detail on how to interpret the significance of the features and the effect of their weights on the prediction of KAM reduction (lines 289-308). We believe that the discussion of feature salience and our proactive interpretation of the regression coefficients will help the interested reader better understand the significance of the feature set and apply these results to their own work.

“Holding all other inputs constant, the model’s sensitivity to a 12° change toward a more valgus alignment changed the mean KAM peak reduction to less than 0.50 %BW*HT, which has previously been used as the clinically meaningful threshold related to risk of disease progression [6, 11, 28]. The amplitude of this reduction is less than the 16° valgus change found to persist six months after a high tibial osteotomy surgery [24], suggesting that the model’s sensitivity is physiologically feasible and that static alignment is a relevant predictor for KAM reduction.”

“Here, the model’s sensitivity to an added 40 kg resulted in a mean KAM peak reduction that was below the clinically meaningful threshold; at an average height of 170 cm, this added weight would increase BMI from 21 to 35, which is the threshold for obesity [36]. Since knee osteoarthritis patients with a higher weight have a larger weight normalized KAM than their lean age matched osteoarthritic controls [37], further investigation is warranted to determine why individuals at a higher weight may be unable to achieve significant reductions to KAM despite their larger KAM.”

We believe that these additions provide important context to our results that can increase the applicability of the predictive model.

4. Similarly to the Introduction, the Discussion would benefit from the comparison with the state-of-the-art in data-driven approaches, clearly stating how this research advances compared to similar studies.

We have clarified in lines 255-256 that our data-driven approach predicts KAM reduction after a gait retraining, instead of estimating KAM from gait data:

“The aim of this work was to build a predictive model that uses minimal clinical data to estimate reduction in the first peak of KAM during a toe-in gait retraining.”

A model to predict KAM reduction has not been previously reported in the literature. Another novel aspect of our study is that we trained the KAM reduction model on synthetic data and showed that it generalizes to real data. In addition to the proposed model targeting a new task, a two-step validation of this kind has not been reported in prior studies. We have reinforced the novelty of our work and distinction from prior work in lines 258-260:

“This validation on independent data, in addition to the traditional approach of validating on left-out test data, gives us cautious optimism in the model’s generalizability.”

**Additional changes:**

We would like to bring to your attention that we have removed the sections reporting LASSO regularization from this manuscript. This change does not affect the results in any way. We have made this decision after a reappraisal of whether this method is appropriate for our work, finding that LASSO is most helpful in avoiding overfitting via shrinkage and regularization when the feature space is high; this was not the case here, as our training matrix has 1104 rows for 6 features. We believe this change can help to avoid potential confusion for the readers. Further detail on when it is appropriate to regularize with LASSO can be found in the original paper by Tibshirani (1996).

As we handled this revision, we also carefully re-edited the entire manuscript to increase clarity and consistency. These changes are all marked in the highlighted version of the revised manuscript.

Finally, we made stylistic changes to all the figures to make them more consistent with one another.
